# Contributions of Root WSC during Grain Filling in Wheat under Drought

**DOI:** 10.3389/fpls.2016.00904

**Published:** 2016-06-23

**Authors:** Jingjuan Zhang, Bernard Dell, Wujun Ma, Rudy Vergauwen, Xinmin Zhang, Tina Oteri, Andrew Foreman, Damian Laird, Wim Van den Ende

**Affiliations:** ^1^School of Veterinary and Life Sciences, Murdoch University, MurdochWA, Australia; ^2^Laboratory of Molecular Plant Biology, KU LeuvenLeuven, Belgium; ^3^School of Engineering and Information Technology, Murdoch University, MurdochWA, Australia

**Keywords:** 6-kestose, deep rooting, fructan remobilization, grain assimilation, osmotic adjustment, root water soluble carbohydrates (WSC), terminal drought stress

## Abstract

As the first organ in plants to sense water-deficit in the soil, roots have important roles for improving crop adaption to water limited environments. Stem water soluble carbohydrates (WSC) are a major carbon source for grain filling under drought conditions. The contributions of root WSC during grain filling under drought has not been revealed. Wheat parental lines of Westonia, Kauz and their derived four double haploid (DH) lines, namely, DH 125, DH 139, DH 307, and DH 338 were used in a field drought experiment with four replications. Through measurements of the root and stem WSC components, and the associated enzyme activities during grain filling, we identified that the levels of root WSC and fructan were one third of the levels in stems. In particular, root glucose and 6-kestose levels were one third of the stem, while the root fructose and bifurcose level were almost half of the stem and sucrose level was two third of the stem. The accumulation and the degradation patterns of root fructan levels were similar to that in the stem, especially under drought. Correlations between root fructan levels and grain assimilation were highly significant, indicating that under terminal drought, root WSC represents a redistributed carbon source for grain filling rather than deep rooting. The significantly higher root sucrose levels under drought suggest that sucrose may act as a signal under drought stress. As compared with stem fructose levels, the earlier increased root fructose levels in DH 307, DH 139, and DH 338 provided agile response to drought stress. Our root results further confirmed that β-(2–6) linkages predominate in wheat with patterns of 6-kestose being closely correlated with overall fructan patterns. Further research will focus on the roles of 6-FEH during fructan remobilization in stems.

## Introduction

Along with global warming, drought is considered to be one of the prime abiotic stresses in the world. For producing sustainable crop yield, drought tolerance is the most desirable trait for breeders. Drought tolerance can be defined in different ways, including drought avoidance, high water use efficiency and growth recovery following rewatering (Passioura, 2012).

In wheat, before flowering, stem, and roots are the major sinks (Xue et al., 2008; Paul and Lawlor, 2014). Plant survival during the drought conditions can be supported by osmotic adjustment. Most of the adjustment can usually be resulted by increases in concentration of common solutes, including sugars, organic acids, amino acids, and inorganic ions (especially K^+^). Some results show that dehydration tolerance can be promoted by osmotic adjustment, but this does not always lead to higher productivity (McCree and Richardson, 1987). Plant turgor and root growth can be maintained at lower water potentials. Drought stress enhances root extension into deeper, moist soil (Fukai and Cooper, 1995). Thus, varieties with deep roots become an important practical way to select for drought tolerance (Fukai and Cooper, 1995; Price et al., 1997; Wasson et al., 2012). However, root elongation is limited by soil impedance. Deep rooting is significantly limited while penetrometer resistances are more than 2 MPa, air-filled volume is less than 10%, and a matric potential is higher than -1.5 MPa (Bengough et al., 2011; Lynch and Wojciechowski, 2015). Root growth is reduced and stressed roots develop a pronounced suberisation of the apoplast to minimize water losses for plant survival (Steudle, 2000).

When drought occurs during the reproductive and grain filling stages of wheat, general responses including stomatal closure, photosynthesis limitation, osmotic adjustment, abscisic acid (ABA) accumulation, and root elongation would occur. Male sterility is a particular phenotype during such process (Blum, 2007). When drought stress becomes severe, the phloem translocation mechanisms may be affected directly, since phloem transport relies on water transport processes in the xylem. However, it was demonstrated that phloem translocation remained unaffected until late in the stress period, when other processes, such as photosynthesis, had already been strongly inhibited (Taiz and Zeiger, 2002). Because of this relative insensitivity of translocation to stress, plant reserves can be mobilized, for example, to grain. The continuing translocation of assimilates could be a key ability for drought tolerance.

Under drought stress, stem water soluble carbohydrate (WSC), mainly fructans, represents a long-term carbon storage form functioning as a major carbon source for grain filling (Pollock, 1986; Pollock and Cairns, 1991). The high remobilization efficiency of stem WSC could contribute to high water use efficiency (Passioura, 2012; Zhang et al., 2015a). Fructans may also be involved in osmoregulation under drought (Schnyder, 1993; Turner et al., 2008). Stem fructans may also contribute to recovery mechanisms under biotic and abiotic stresses (Schnyder, 1993; Yang and Zhang, 2006; Valluru and Van den Ende, 2008; Livingston et al., 2009). Furthermore, sucrose, hexoses (Hex) and small fructans may act as phloem-mobile signaling molecules under stress, contributing to stress tolerance and disease prevention (Van den Ende, 2013; Ruan, 2014).

There are different fructan types in wheat, predominantly graminan- and levan-type fructans in which β-(2–6) linkages predominate, besides some small inulin-type fructans (Pollock and Cairns, 1991). 1-SST (sucrose: sucrose 1-fructosyl transferase), 1-FFT (fructan: fructan 1-fructosyltransferase), and 6-SFT (sucrose: fructan 6-fructosyltransferase) are the main fructosyltransferases involved. Fructans are degraded into sucrose and fructose by linkage-specific fructan exohydrolases, for example, 1-FEH, 6-FEH, and 6&1-FEH (Van den Ende et al., 2003; Kawakami et al., 2005; Van Riet et al., 2006, 2008; Xue et al., 2008). Sucrose is hydrolyzed to Hex through different types of invertases (INVs), among which soluble acid-type vacuolar invertase is the most prominent form in wheat roots (Königshofer and Löppert, 2015).

Carbohydrate metabolism has previously been studied in wheat roots under cold and salinity stresses (Santoiani et al., 1993; Kafi et al., 2003). Chilling stimulated high degree of polymerization (DP) fructan synthesis associated with higher sucrose levels and higher 1-SST and sucrose phosphate synthase (SPS) activities whereas INV and FEH activities remained unaffected. Cold temperature decreased shoot and root growth and increased carbohydrate levels in different wheat cultivars (Equiza et al., 1997), as it does in many other species (Tarkowski and Van den Ende, 2015). In ryegrass, lines adapted to cold climates produced more high DP fructans as compared to lines adapted to warmer environments when subjected to cold stress (Abeynayake et al., 2015). Under salt stress, contents of proline, soluble and insoluble carbohydrates increased in leaves, apices and roots but were lower in the maturing seeds of a salt-sensitive wheat cultivar (cv. Ghods) as compared to salt tolerant cultivars (Kafi et al., 2003).

So far, wheat stem WSC dynamics have been well studied under an array of stresses. However, carbohydrate dynamics in drought-stressed wheat roots remains unknown. At grain filling stage, the three hypotheses for contributions of root WSC would be: (i) as an energy source for root growing deeper to utilize the moisture in deep soil; (ii) as an osmoregulation factor for maintaining plant vigor and keep plant survive; and (iii) as a redistributed carbon source for grain filling. To better understand the root responses to drought at the grain filling stage, glucose, fructose, sucrose and different types of fructans were examined in wheat roots alongside stems under different water regimes. Enzyme activities involved in fructan biosynthesis and degradation along with vacuolar invertase activity were analyzed in parallel, with the objective to: (i) understand the potential function of root WSC and (ii) identify key enzymes during the process.

## Materials and Methods

### Plant Materials

Wheat varieties, Westonia, Kauz and their double haploid (DH) lines DH 125, DH 139, DH 307, and DH 338 were selected from a population of 225 lines and used in a field drought experiment. Westonia and Kauz were developed in Western Australia (WA) and the International Maize and Wheat Improvement Center (CIMMYT, Mexico), respectively. Westonia produces consistent high yield in medium and low rainfall regions as Kauz is considered as drought tolerant variety (Butler et al., 2005). Both varieties contain high stem WSC levels (∼40%) after anthesis but they show different responses to the drought stress as explained previously (Zhang et al., 2009, 2015b). DH 125, DH 139, DH 307, and DH 338 were selected based on genetic diversity and flowering time (Zhang et al., 2015b). Previous results showed that DH 139 and DH 307 are genetically close to Westonia while DH 125 and DH 338 are close to Kauz. DH 139 contains the 1-FEH w3 Westonia type of allele while the three other DH lines contain the Kauz type of 1-FEH w3 alleles (Zhang et al., 2015b). DH 307 and DH 125 flower at a similar time, while DH 139 and DH 338 flower relatively later. Under drought, the losses in the grain weight per spike in DH 307, DH 125, and DH 338 were less than in DH 139 (data not shown).

### Field Experiment

The field drought trial was carried out in 2013 at Merredin field station, Western Australia (31.5° S, 118.3° E). Parental lines of Westonia and Kauz and DH 125, DH 139, DH 307, and DH 338 were planted in 10 m^2^ plots, in a randomized trial with four replicates sown on the 20th of June for both drought and irrigated treatments. Drought treatment was initiated at average anthesis time (the 24th of September, 2013) of these lines. Besides 16 mm of rainfall (6 and 10 mm rainfall on the 9–10th and 20–21st of October, 2013), irrigated plots received 15 mm water on a weekly basis for four weeks after the average anthesis time. Twelve neutron probes (down to 1.5 m depth) were distributed evenly in each treatment block to monitor soil moisture. During the drought treatment, soil water content was significantly reduced by 40% at 10 cm depth and reduced by 15 and 8% at 30 and 50 cm depth, respectively, in the late sowing trial between 20 and 30 days after anthesis (DAA) (**Supplementary Figure S1**).

### Plant Harvest

Six plants of each plot were sampled weekly between 11:00 and 17:00 (Zhang et al., 2008) from 1-week pre-anthesis to 6-weeks post-anthesis. A shovel was used to dig out the plants, including almost their entire root system above 20–30 cm depth. Plants were separated from each other after the soil was gently removed. The six main stems of the six plants were immediately placed on dry ice and subsequently stored in a -20°C freezer. The stem samples for the WSC extraction were processed as described previously (Zhang et al., 2008). Roots from the six plants in each plot were combined to provide one root sample per plot. Roots were rinsed free of soil on a sieve with 2 mm apertures, immediately surface dried using tissue paper then the root fresh weight was recorded. The roots were stored (-20°C) afterwards. Once frozen, each root sample was ground to fine powder using a TissueLyser II (Qiagen) in a 4°C temperature controlled room. Samples were then subdivided for enzyme analysis, and WSC and WSC components analysis, as described previously (Zhang et al., 2015a). The six heads were combined and stored (-20°C). Four out of six heads were randomly selected, freeze dried and oven dried. Seeds were taken by hand threshing for grain weight (GW) assimilation measurements. Data were generated weekly, including thousand grain weight (TGW), kernel number per spike (KN), and grain weight (GW) per spike from main stems. At harvest time, 1 m^2^ of each plot was harvested and grain weight (GW) per m^2^, GW per tiller, KN per tiller, and TGW were recorded.

### Carbohydrate Analysis

Total WSC were extracted from stem and root powder using the deionized water and anthrone reagent (Fales, 1951; Yemm and Willis, 1954). The WSC content was analyzed as previously described (Zhang et al., 2015b). Briefly, WSC components were separated by high-performance anion exchange chromatography with integrated pulsed amperometric detection (HPAEC–IPAD) and quantified using peak area comparison. The glucose, fructose, sucrose, 1-kestose, 6-kestose, neokestose, nystose, and bifurcose were used as external standards. Total fructan concentration was calculated as described previously (Zhang et al., 2015a).

### Enzyme Activity Measurements

For reducing the high overall work load, the root samples of Westonia, Kauz, DH 125 and DH 307 were selected for sugar related enzyme activity measurements. Protein extraction and enzyme activity measurements were undertaken as described previously (Zhang et al., 2015b).

### Statistical Analysis

Analysis of variance (ANOVA) in IBM SPSS statistics v 21 was used for phenotype data analysis. Significant groupings were identified by *post hoc* Tukey’s Multiple Range tests. The data pair significant analysis was detected by the Student’s *t*-test. The correlation significance level was determined by Pearson bivariate analysis.

## Results

### Root Fructan and Grain Assimilation

Under drought, grain yield losses per square meter were only significant for the late flowering lines DH 139 and DH 338 (**Supplementary Table S1**). The reduction in grain weight per tiller under drought was significant in DH 307 and DH 139. The KN per tiller was only significantly affected in DH 139 under drought. A significant TGW loss appeared in most lines, with the exception of Kauz and DH 139.

**FIGURE 1:**
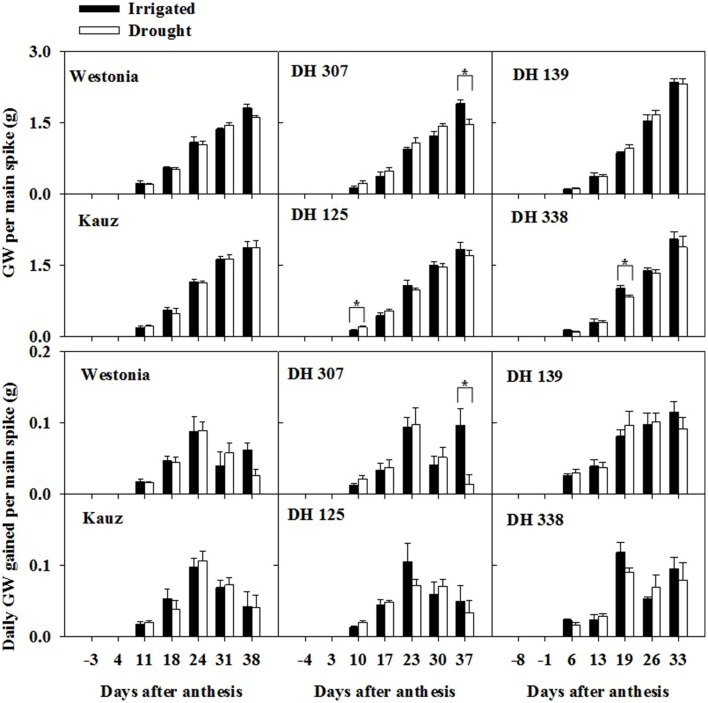
**Time-dependent grain weight per main spike and daily grain weight gains for DH 307, DH 125, DH 139, DH 338, and the parental lines of Westonia and Kauz under irrigated and drought conditions.** The vertical bars represent SE. An asterisk (^∗^) identifies significantly different values at *P* < 0.05 in *t*-test

The grain assimilation patterns from the main stem were different among lines and treatments. Under drought, the daily grain weight (GW) peaked around 23–24 DAA before decreasing, except for DH 139 and DH 338 (**Figure 1**). In irrigated plants, the daily assimilation rate patterns were similar between Kauz and DH 125 and similar under drought, while the other lines still showed enhanced rates towards the end of the sampling period under irrigated conditions. The daily GW assimilation was significantly higher in DH 307 irrigated plants at the last sampling time as compared to that under drought (**Figure 1**), which may account for the significantly higher GW per main spike and final GW per tiller under irrigated conditions (**Figure 1**; **Supplementary Table S1**).

**FIGURE 2:**
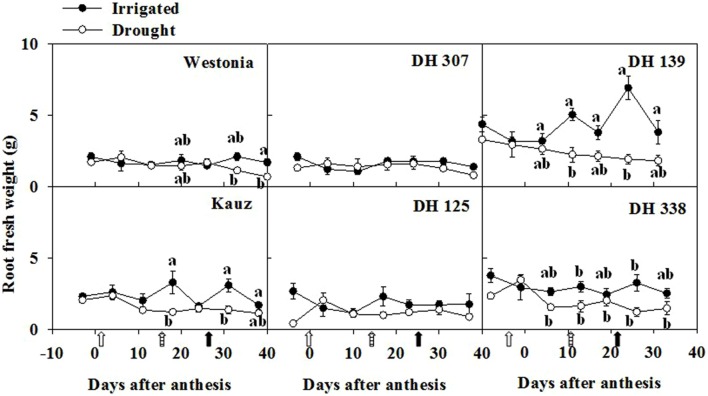
**Time-dependent root fresh weight of six plants for DH 307, DH 125, DH 139, DH 338, and the parental lines of Westonia and Kauz under irrigated and drought conditions.** The vertical bars represent SE. Values with the same letter are statistically not different at *P* = 0.05. Open, dashed and closed arrows indicate start of drought treatment, 6 and 10 mm of rainfall, respectively.

As root growth relies on the sucrose supplies from photosynthesis, root WSC levels may reflect root development under different water regimes. Root fresh weight significantly increased in Kauz and DH 139 under irrigated conditions (**Figure 2**). The root fresh weight differences between irrigated and drought conditions were less prominent in the other lines. The highest WSC level in roots was around 10–15% of dry weight (DW), which was approximately one third of the level recorded in stems (30–45% of DW) (**Supplementary Figure S2**). Between 25 and 30 DAA, WSC concentration in roots decreased significantly in all lines under drought. These significant differences were maintained up to the last sampling date in some lines (DH 307, DH 139, and Westonia; **Supplementary Figure S2**).

**FIGURE 3:**
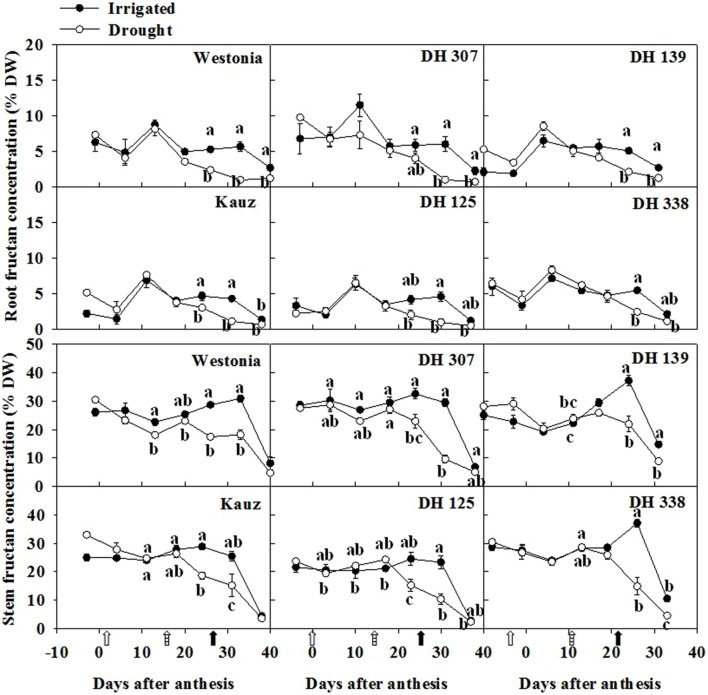
**Fructan levels in roots and stems of DH 307, DH 125, DH 139, DH 338, and the parental lines of Westonia and Kauz under drought and irrigated conditions in the field, respectively.** The vertical bars represent SE. Values with the same letter are statistically not different at *P* = 0.05. Open, dashed and closed arrows indicate start of drought treatment, 6 and 10 mm of rainfall, respectively.

As fructans are the major storage form of stem WSC (Pollock and Cairns, 1991; Zhang et al., 2015a), the root fructan level was examined. The maximal root fructan concentration was around 7–10% of DW (**Figure 3**). Similar to root WSC, the root fructan level was about one third of the stem fructan level (25–30% of DW) and it decreased significantly after 20 DAA in all lines. This implies that the concentrations of WSC in roots and stems are inter-related and statistical analysis showed a high significant correlation (*P* < 0.01) in Kauz, DH 125, and DH 338 under both water regimes (**Supplementary Figure S3**). It was well correlated in DH 307 drought plants (*R*^2^ = 0.53^∗∗^, *P* < 0.01) while the correlation was lower (*R*^2^ = 0.26^∗^, *P* < 0.05) under irrigated conditions.

**FIGURE 4:**
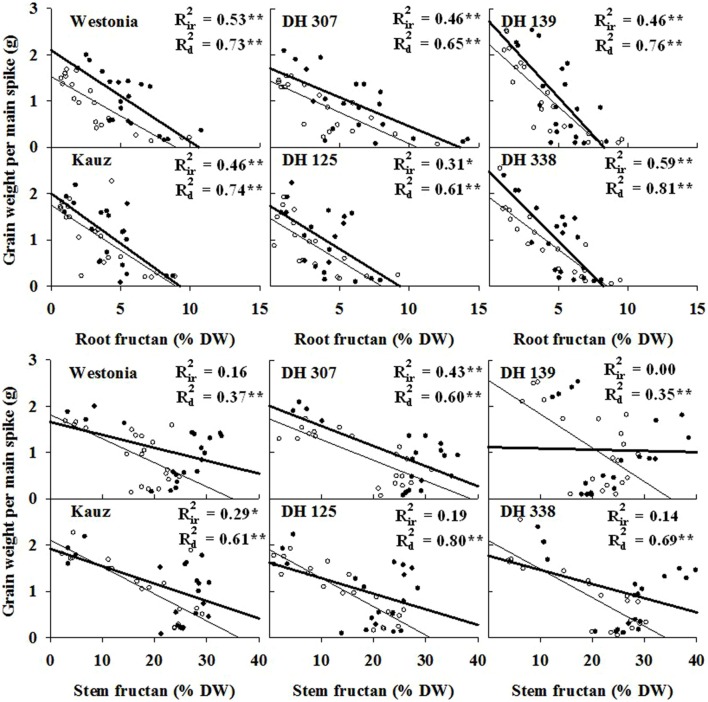
**The correlations of grain weight per main spike and fructan concentration in roots and stems (sheath included) starting from six days after anthesis (DAA) in DH 307, DH 125, DH 139, DH 338, and the parental lines of Westonia and Kauz under irrigated (closed circles and thick lines) and drought conditions (open circles and thin lines), respectively.** *R*_ir_^2^ and *R*_d_^2^ represent the correlations under irrigated and drought conditions, respectively. Asterisks (^∗^) and (^∗∗^) represent the significant levels at *P* < 0.05 and *P* < 0.01, respectively.

Since fructans are recognized sources for grain filling, the association between root fructan degradation and grain assimilation was examined. Significant correlations (*P* < 0.01) between grain weight and fructan level were detected under both water regimes in all lines (**Figure 4**). Overall, correlations were superior under drought as compared to irrigated conditions (**Figure 4**). Moreover, with the exception of DH 125 under drought, correlative parameters were even better for root fructans as compared to stem fructans (**Figure 4**).

**FIGURE 5:**
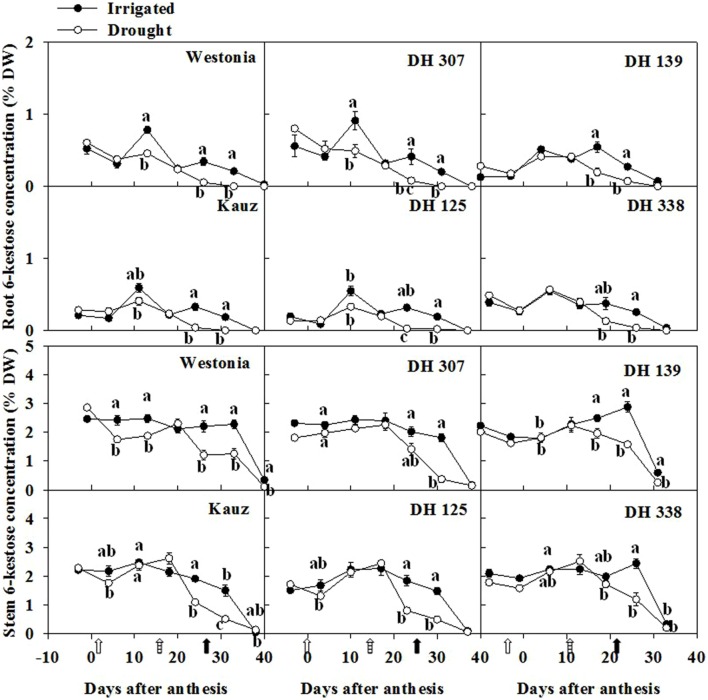
**The levels of 6-kestose in roots and stems of DH 307, DH 125, DH 139, DH 338, and the parental lines of Westonia and Kauz under drought and irrigated conditions in the field, respectively.** The vertical bars represent SE. Values with the same letter are statistically not different at *P* = 0.05. Open, dashed and closed arrows indicate start of drought treatment, 6 and 10 mm of rainfall, respectively.

Similar to total fructan levels, the 6-kestose level in roots was almost one third of that in stems. After anthesis, 6-kestose increased in irrigated plants up to about 10 DAA and decreased gradually afterwards (**Figure 5**). Under drought, only a slight increase occurred, followed by a significant decrease between 20 and 30 DAA in Westonia, Kauz, DH 307, and DH 125 (**Figure 5**). In the stem, 6-kestose levels became significantly lower under drought in all DH lines between 20 and 30 DAA (**Figure 5**). Overall, root and stem 6-kestose correlated well with root and stem total fructans (**Supplementary Figure S4**). The bifurcose level in roots was almost half of the level in stems (**Supplementary Figure S5**). The bifurcose patterns resembled those of 6-kestose, with some line-specific variations (**Figure 5**; **Supplementary Figure S5**). Levels of 1-kestose and 1,1-nystose in roots were hardly detectable. In stems, 1-kestose levels were below 1% in all lines and much lower than 6-kestose and bifurcose, decreasing slightly in all lines and under both treatments (data not shown).

### Fructose, Glucose, and Sucrose Dynamics

**FIGURE 6:**
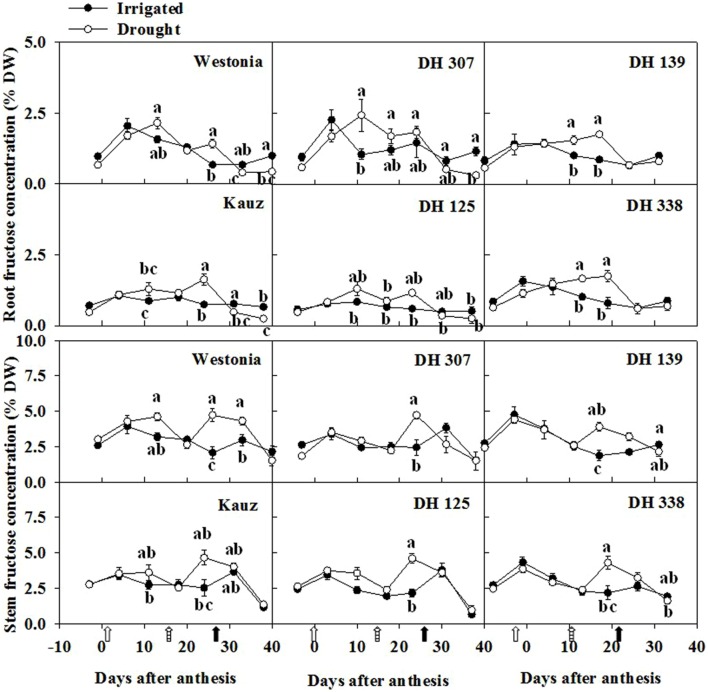
**The levels of fructose in roots and stems of DH 307, DH 125, DH 139, DH 338, and the parental lines of Westonia and Kauz under drought and irrigated conditions in the field, respectively.** The vertical bars represent SE. Values with the same letter are statistically not different at *P* = 0.05. Open, dashed and closed arrows indicate start of drought treatment, 6 and 10 mm of rainfall, respectively.

**FIGURE 7:**
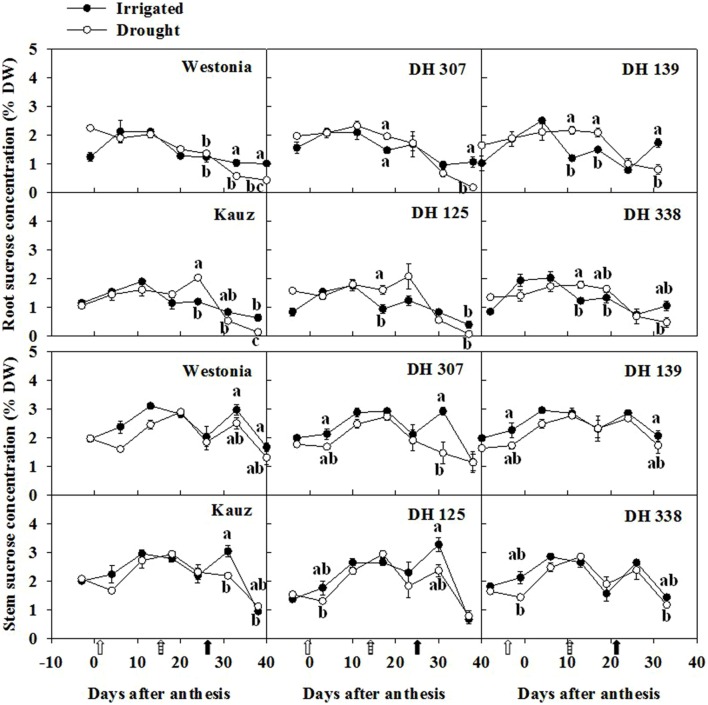
**The levels of sucrose in roots and stems of DH 307, DH 125, DH 139, DH 338, and the parental lines of Westonia and Kauz under drought and irrigated conditions in the field, respectively.** The vertical bars represent SE. Values with the same letter are statistically not different at *P* = 0.05. Open, dashed and closed arrows indicate start of drought treatment, 6 and 10 mm of rainfall, respectively.

Along with the root fructan degradation, the fructose, glucose and sucrose were examined. In general, fructose levels in roots were half of the levels in stems. For all lines, except for DH 125, there were one or two dates at which the root fructose levels became significantly higher under drought than under comparable irrigated conditions (**Figure 6**). With the exception of Kauz, differences in stem fructose levels between drought and irrigated plants were more extended. The early increase of fructose in DH 307 roots did not appear in stems. The glucose levels in roots were approximately one third of the levels in stems. For all lines, except for DH 125, DH 307, and Westonia, there were one or two dates at which the root glucose levels became significantly higher under drought (**Supplementary Figure S6**). In drought treated plants, the root glucose level in DH 307 was significantly higher than that in DH 125. In stems, glucose levels were significantly higher under drought, at least at one point in time, for all lines (**Supplementary Figure S6**). After 10 DAA, patterns of glucose and fructose were very similar (**Figure 6**; **Supplementary Figure S6**). In general, root sucrose levels were only slightly lower than those in stems (**Figure 7**). Interestingly, root sucrose levels under drought became significantly higher over the 15–20 DAA intervals in all lines, except for DH 307 and Westonia (**Figure 7**).

### Fructan and Sucrose Metabolic Enzymes

**FIGURE 8:**
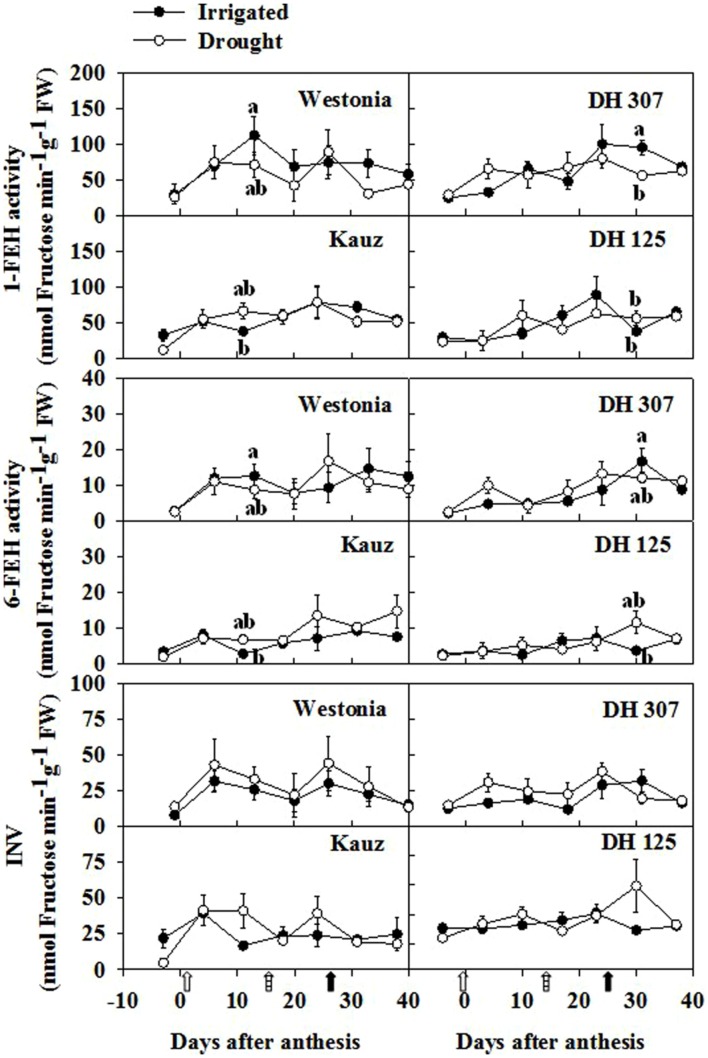
**Enzyme activities of 1-FEH, 6-FEH, and INV at fresh level in roots of DH 307, DH 125, DH 139, DH 338, and the parental lines of Westonia and Kauz under drought (open circles) and irrigated conditions (closed circles) in the field.** Open, dashed and closed arrows indicate start of drought treatment, 6 and 10 mm of rainfall, respectively.

Since fructan biosynthetic and breakdown enzymes influence fructan levels and other WSC components, the activities of the enzymes involved in fructan and sucrose metabolism were investigated. In roots, the enzyme activities of 1-SST, 6-SST, and 1-FFT were extremely low (data not shown), and did not correlate with increasing fructan levels around 10 DAA in most lines. Hydrolytic activities (1-FEH, 6-FEH, and vacuolar invertase) increased in some lines over the sampling period but no significantly higher activities could be detected between drought and irrigated conditions (**Figure 8**). At 10 DAA in irrigated plants, 1-FEH and 6-FEH activities were significantly higher in Westonia than in Kauz. At 30 DAA in irrigated plants, 1-FEH and 6-FEH activities were significantly higher in DH 307 as compared to DH 125 (**Figure 8**).

## Discussion

Wheat stem WSC is recognized as a storage carbon source for grain filling and fructans are the main component (Schnyder, 1993; Yang et al., 2000; Zhang et al., 2015a, b). Equivalent to one third of the stem WSC level, the root WSC may be as a redistributed carbon source pool for grain filling. Thus, the decrease of root WSC may be associated with the grain assimilation rate as indicated by Lopes and Reynolds (2010). Root fructan and 6-kestose levels peaked around 10 DAA under both water regimes. Afterwards, they decreased significantly under drought. During wheat grain development, the basic structure of the endosperm is established after 10 DAA and thereafter, the deposition of storage components (starch and proteins) is initiated (Shewry et al., 2012). The rapid grain filling period is between 14 and 28 DAA (Shewry et al., 2012). According to our data, the most active grain filling period was between 18 and 24 DAA (**Figure 1**). For late flowering lines, this period extended to 33 DAA (**Figure 1**). The period of rapid grain filling correlated well with the decrease in the root WSC level. The likely remobilization of the root WSC to grain was also strongly supported by the significant correlation between root fructan level and GW assimilation. The degradation of root fructan was more distinct under drought, indicating that root fructan remobilization was more efficient than under irrigated conditions.

Fructans in wheat include β-(2–1) and β-(2–6) linkages and the latter is the predominant form (Bancal et al., 1993; Bonnett and Simpson, 1995; Van den Ende et al., 2003). From our results, the patterns of 6-kestose and total fructan were very similar in the stems and roots. The correlations between 6-kestose and total fructan were highly significant in both stems and roots under two water regimes. Our results further support that β-(2–6) linkages are the predominant form of fructan in wheat.

Studies under controlled environments in wheat (Santoiani et al., 1993; Equiza et al., 1997; Kafi et al., 2003) have demonstrated that levels of fructan and monosaccharides accumulate in roots under cold and salinity stresses. By contrast, our study was conducted under field conditions, with wheat plants going through natural drought. In contrast to the above-mentioned results, root fructan levels decreased significantly under drought in all lines. The differences are probably due to the growth stages at sampling and the related different function of the root WSC. In the previous studies (Santoiani et al., 1993; Equiza et al., 1997; Kafi et al., 2003), the root samples were taken before flowering. Our drought stress commenced at flowering and the root samples were taken during grain filling. It seems that plants tend to accumulate more WSC in root under abiotic stress before anthesis and use mechanisms of the osmotic adjustment for surviving. At the grain filling stage, although in the process of the root fructan degradation, the significant high levels of sucrose, fructose and glucose in drought plants would act as osmoregulators, the main function of root WSC may be as partial fructan pools for translocation to grain, as growing seeds is a priority for plants during terminal drought.

A wheat growth study on four soil types in WA revealed that wheat roots grew more rapidly during seedling and anthesis stages, while growth rates reduced during tillering and grain filling stages (Tennant, 1976; Watt et al., 2013), suggesting that tillering and grain filling are more costly (in terms of energy/carbon sources) as compared with root growth. Ideally, the ability of deep rooting allows plants to reach moisture from deeper soil layers to produce relatively high grain yields in dry environments. Indeed, root depths (sampled between 7 and 12 DAA) were positively correlated with wheat yield under terminal or intermittent drought (Lopes and Reynolds, 2010). However, root elongation is affected by soil type, the level of water deficiency and soil compaction (Bengough et al., 2011; Lynch and Wojciechowski, 2015). Moreover, under severe and persistent drought stress, root growth is reduced. For minimizing water losses, stressed roots develop a suberized interface between living tissue and the rhizosphere (Steudle, 2000). Consequently, under severe drought, apart from as an energy source to develop suberisation, the major contributions of root WSC reserves is most likely to serve as a carbon source for grain filling rather than for deep rooting. In this study, weather was not perfect for the drought experiment. There were two rain falls (6 mm on the 9–10th of October and 10 mm on the 20–21st of October) and the evaporations on those days were 4.2 and 5.8 mm, respectively. Apart from the evaporation, the little moisture from the 6 mm rainfall mainly stayed on the soil surface and was probably not enough to help plants develop deep roots. The 10 mm rainfall happened at the late grain fill stage. Apart from the high evaporation (5.8 mm), the rest moisture mainly remained on the top layer of the soil and might favor the grain assimilation in the late flowering lines (DH 139 and 338). It seems very unlikely that this moisture would have helped root elongation instead of grain filling. Since grain filling is clearly the dominant physiological process during these late developmental stages (Tennant, 1976; Lopes and Reynolds, 2010).

Levels of sucrose are proportionally higher in roots, its concentration being two thirds of that in the stem, while the concentrations of other sugars are much lower than those in the stem. Continuous import of leaf-derived photosynthetic sucrose is required to sustain root growth (Nagel et al., 2006), since root carbohydrate reserves cannot sustain root growth for a long time (Eliasson, 1968). During drought stress, photosynthesis can be affected (Zhang et al., 2009), and general growth is affected more than photosynthesis (Vandoorne et al., 2012), explaining why significantly higher levels of sucrose remained in four out of six lines under drought as compared to irrigated plants. In irrigated plants, the lower levels of sucrose in Kauz and DH 139 correlated with higher root fresh weights (*P* < 0.05), in agreement with high sucrose influxes from the leaves and continuous sucrose hydrolysis to sustain root growth. Genotypic differences in root sucrose levels were observed between Westonia and the other lines, and this requires further investigation.

In summary, the levels of root WSC, fructans, glucose and 6-kestose account for one third of that in stems. Root fructose and bifurcose levels were almost half of the stem and the sucrose level was two thirds of the stem. Different from abiotic stresses studied before anthesis, during the grain filling stage, the degradation patterns of root fructan levels and the significantly high correlations with grain assimilation under drought indicate that the root WSC as a partial carbon source for grain filling rather than deep rooting as growing seeds is the priority under terminal drought. Overall, root WSC may represent a redistributed carbon source for grain filling next to stem WSC. Our results further reflect that fructans with β-(2–6) linkages predominate in wheat and the patterns of 6-kestose resembled those of total fructans. Further research is required into the specific roles of root and stem 6-FEHs under terminal drought.

## Author Contributions

JZ and WV designed the experiments. JZ and XZ sampled the materials. JZ, RV, XZ, AF, and TO analyzed the carbohydrates. JZ and RV analyzed the enzyme activities. JZ, WV, BD, WM, and DL wrote the manuscript with inputs from the other authors.

## Conflict of Interest Statement

The authors declare that the research was conducted in the absence of any commercial or financial relationships that could be construed as a potential conflict of interest.
