# Supplementary material for: Contributions of Root WSC during Grain Filling in Wheat under Drought
Source: Front Plant Sci. 2016 Jun 23;7:904. doi: 10.3389/fpls.2016.00904 (PMC4917532; doi:10.3389/fpls.2016.00904)
Supplement: Supplementary file 1 [file Presentation_1.PDF]

Supplementary Materials:

Contributions of root WSC during grain filling in wheat under drought

Jingjuan Zhang[1,*], Bernard Dell[1], Wujun Ma[1], Rudy Vergauwen[3], Xinmin Zhang[1], Tina Oteri[2], Andrew Foreman[2], Damian Laird[2], Wim Van den Ende[3]

[1] School of Veterinary and Life Sciences, Murdoch University, South Street, Murdoch, WA, Australia; [2] School of Engineering and Information Technology, Murdoch University, South Street, Murdoch, WA, Australia; [3] Laboratory of Molecular Plant Biology, Leuven, Belgium

***Correspondence:**

Jingjuan Zhang
School of Veterinary and Life Sciences,
Murdoch University, 90 South Street,
Murdoch, WA 6150, Australia
J.Zhang@murdoch.edu.au

SUPPLEMENTARY TABLE AND FIGURES

Supplementary Table 1. The average grain weight per square meter and the core grain weight components in selected DH lines and the parental lines of Westonia and Kauz under irrigated and drought conditions in Merredin field station in 2013. Values with the same letter are not statistically different at $p = 0.05$

Wheat lines	Treatments	GW per m^2 (g)	GW per tiller (g)	KN per tiller	TGW (g)
Westonia	Irrigated	499.3 ± 4.1 abcd	1.5 ± 0.04 bcd	34.8 ± 1.0 e	**43.8 ± 0.3 abc**
	Drought	424.4 ± 13.7 cd	1.3 ± 0.07 d	34.4 ± 1.3 e	**36.5 ± 1.9 def**
Kauz	Irrigated	520.9 ± 27.5 abc	1.8 ± 0.04 ab	45.3 ± 1.3 bc	40.6 ± 0.8 bcd
	Drought	444.5 ± 19.0 cd	1.6 ± 0.04 bcd	43.4 ± 0.9 cd	36.4 ± 0.2 def
DH 307	Irrigated	494.6 ± 17.7 abcd	**1.6 ± 0.06 bc**	34.1 ± 1.9 e	**47.9 ± 1.0 a**
	Drought	419.9 ± 31.1 d	**1.3 ± 0.06 d**	31.7 ± 1.7 e	**40.0 ± 0.5 cde**
DH 125	Irrigated	563.0 ± 11 ab	1.7 ± 0.11 bc	37.4 ± 1.9 de	**44.8 ± 0.7 ab**
	Drought	470.7 ± 23.2 bcd	1.4 ± 0.03 cd	36.9 ± 0.7 de	**36.8 ± 1.4 def**
DH 139	Irrigated	**544.6 ± 17.8 ab**	**2.1 ± 0.10 a**	**59.5 ± 2.8 a**	35.8 ± 0.3 ef
	Drought	**416.3 ± 15.4 d**	**1.7 ± 0.07 bc**	**51.2 ± 1.3 b**	32.8 ± 0.6 f
DH 338	Irrigated	**585.7 ± 27.0 a**	1.8 ± 0.08 ab	44.4 ± 1.2 cd	**40.7 ± 1.0 bcd**
	Drought	**472.3 ± 10.8 bcd**	1.5 ± 0.04 bcd	46.3 ± 0.9 bc	**32.8 ± 0.6 f**

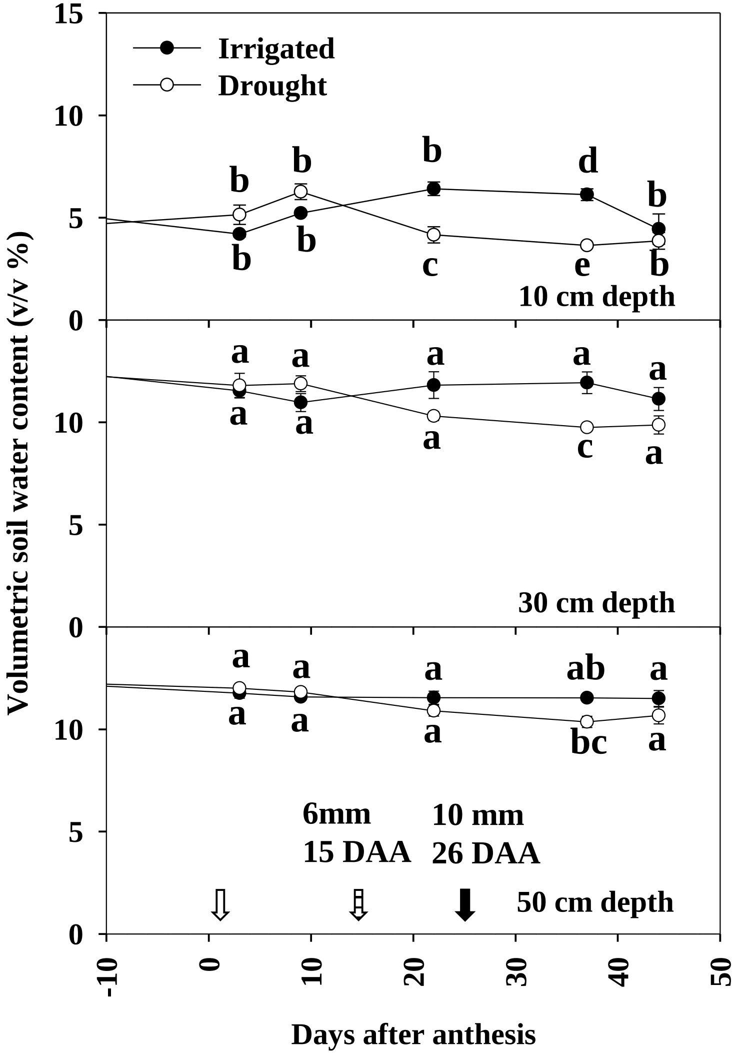

Supplementary Figure S1. Volumetric soil water content (v/v, %) at 10, 30 and 50 cm depth, in drought experiments at Merredin field station in 2013. The average of the days after anthesis of all lines is presented. The vertical bars represent ±SE. Values with the same letter are statistically not different at $p = 0.05$. Open, dashed and closed arrows indicate start of drought treatment, 6 mm and 10 mm of rainfall, respectively

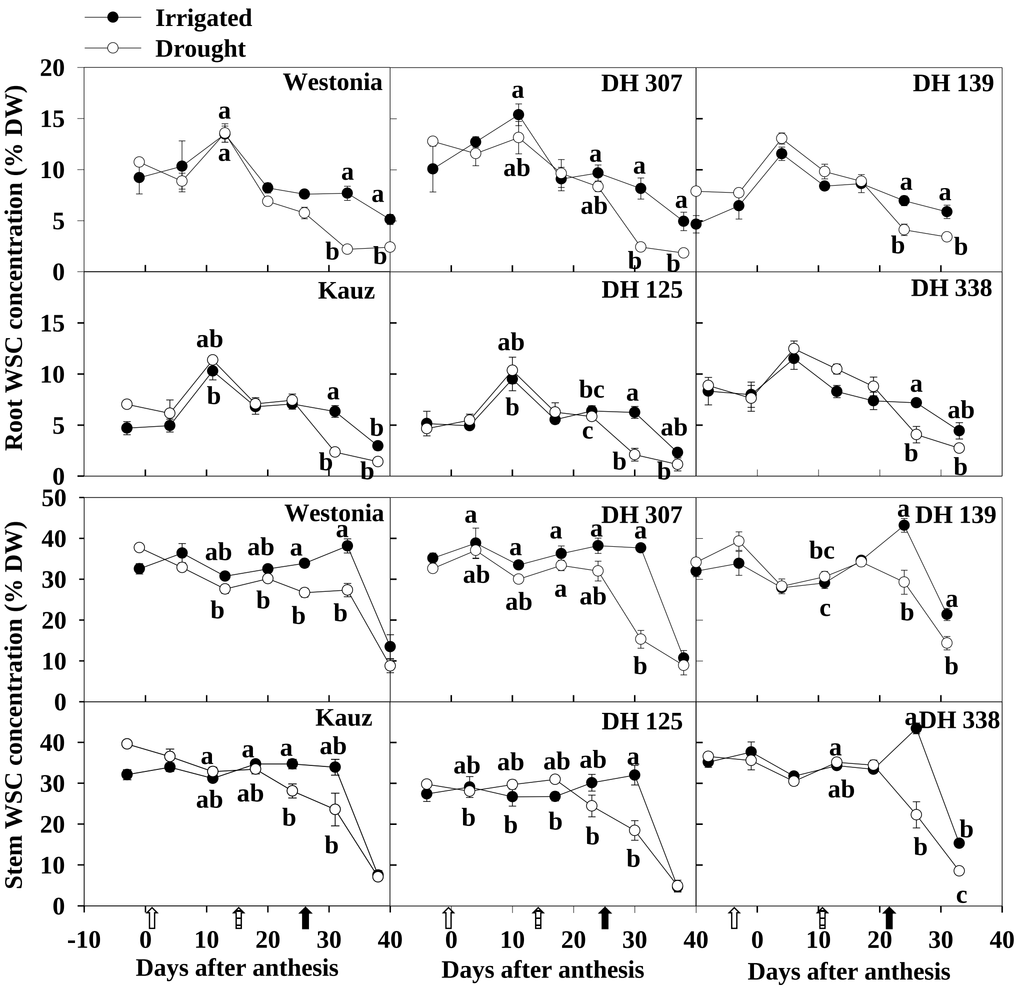

Supplementary Figure S2. Water soluble carbohydrate (WSC) levels in roots and stems of DH 307, DH 125, DH 139, DH 338 and the parental lines of Westonia and Kauz under drought and irrigated conditions in the field. The vertical bars represent SE. Values with the same letter are statistically not different at *P* = 0.05. Open, dashed and closed arrows indicate start of drought treatment, 6 mm and 10 mm of rainfall, respectively

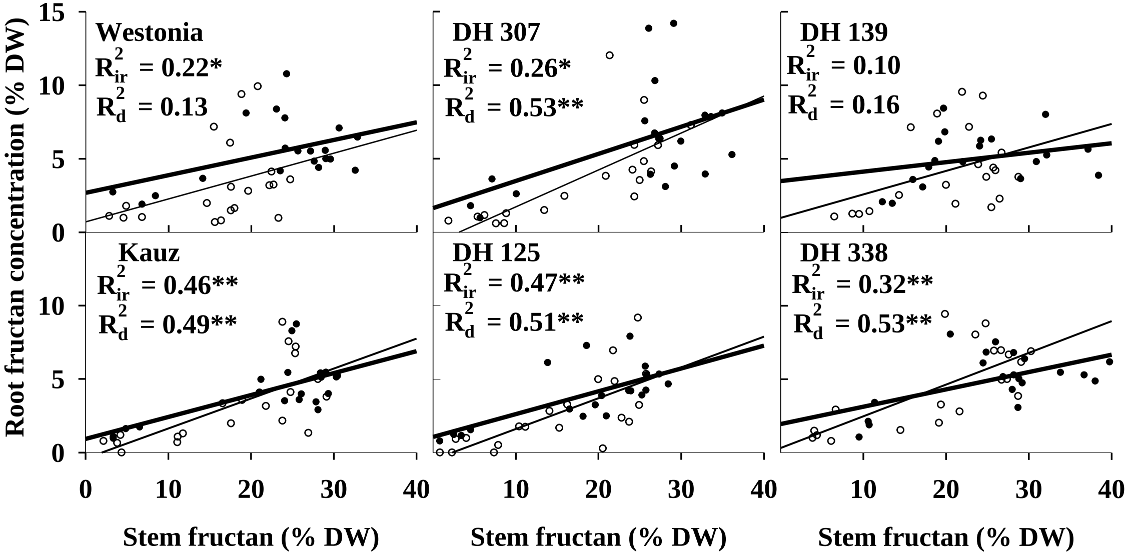

Supplementary Figure S3. The correlations of fructan concentration between stem (sheath included) and root starting from six days after anthesis (DAA) in DH 307, DH 125, DH 139, DH 338 and the parental lines of Westonia and Kauz under irrigated (closed circles, thick lines) and drought conditions (open circles, thin lines). Asterisks (*) and (**) represent the significant levels at P<0.05 and P<0.01, respectively

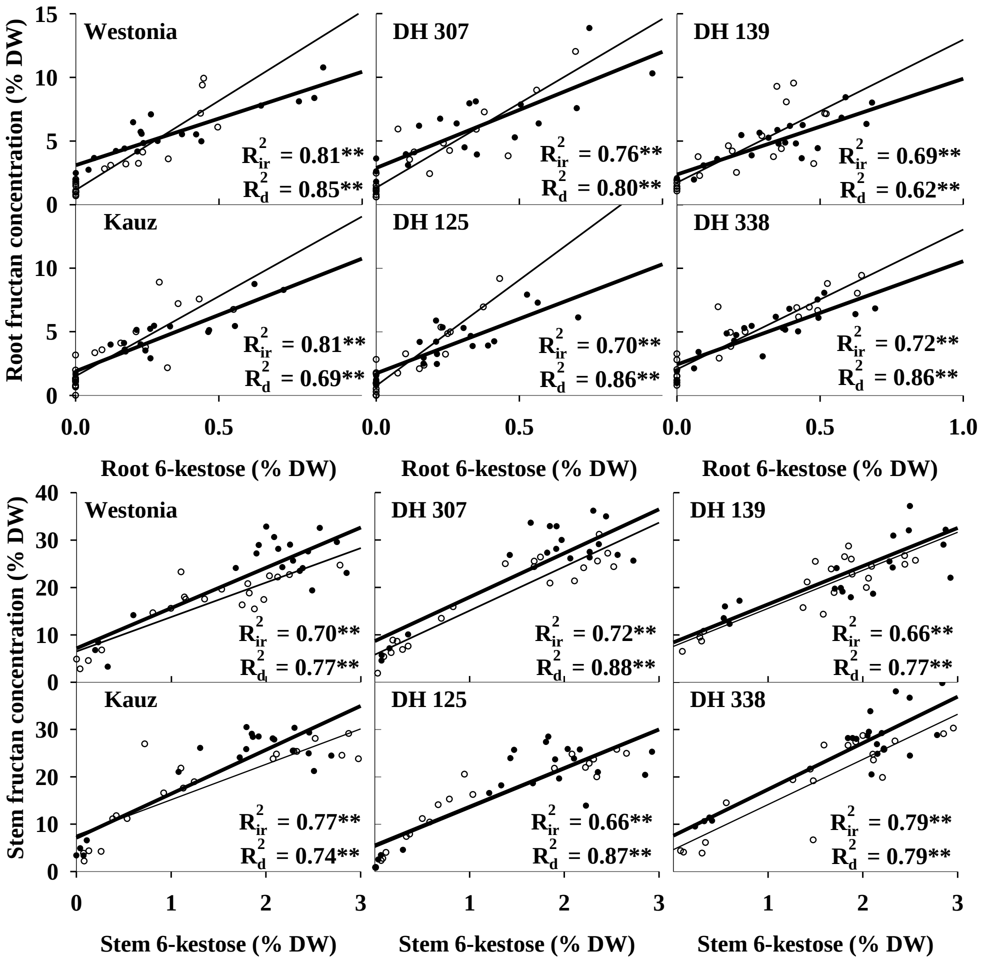

Supplementary Figure S4. The correlations of fructan and 6-kestose concentration in roots and stems (sheath included) starting from six days after anthesis (DAA) in DH 307, DH 125, DH 139, DH 338 and the parental lines of Westonia and Kauz under irrigated (closed circles, thick lines) and drought conditions (open circles, thin lines). Asterisks (*) and (**) represent the significant levels at P<0.05 and P<0.01, respectively

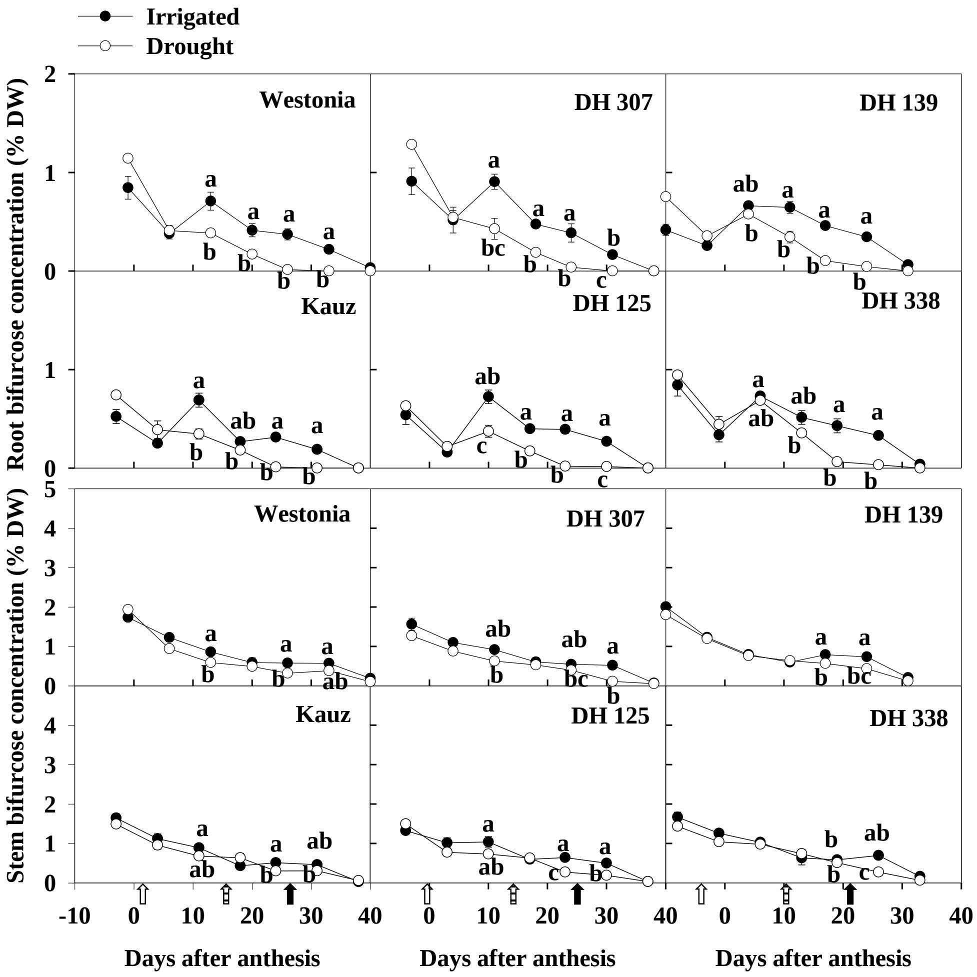

Supplementary Figure S5. Bifurcose levels in roots and stems of DH 307, DH 125, DH 139, DH 338 and the parental lines of Westonia and Kauz under drought and irrigated conditions in the field. The vertical bars represent SE. Values with the same letter are statistically not different at *P* = 0.05. Open, dashed and closed arrows indicate start of drought treatment, 6 mm and 10 mm of rainfall, respectively

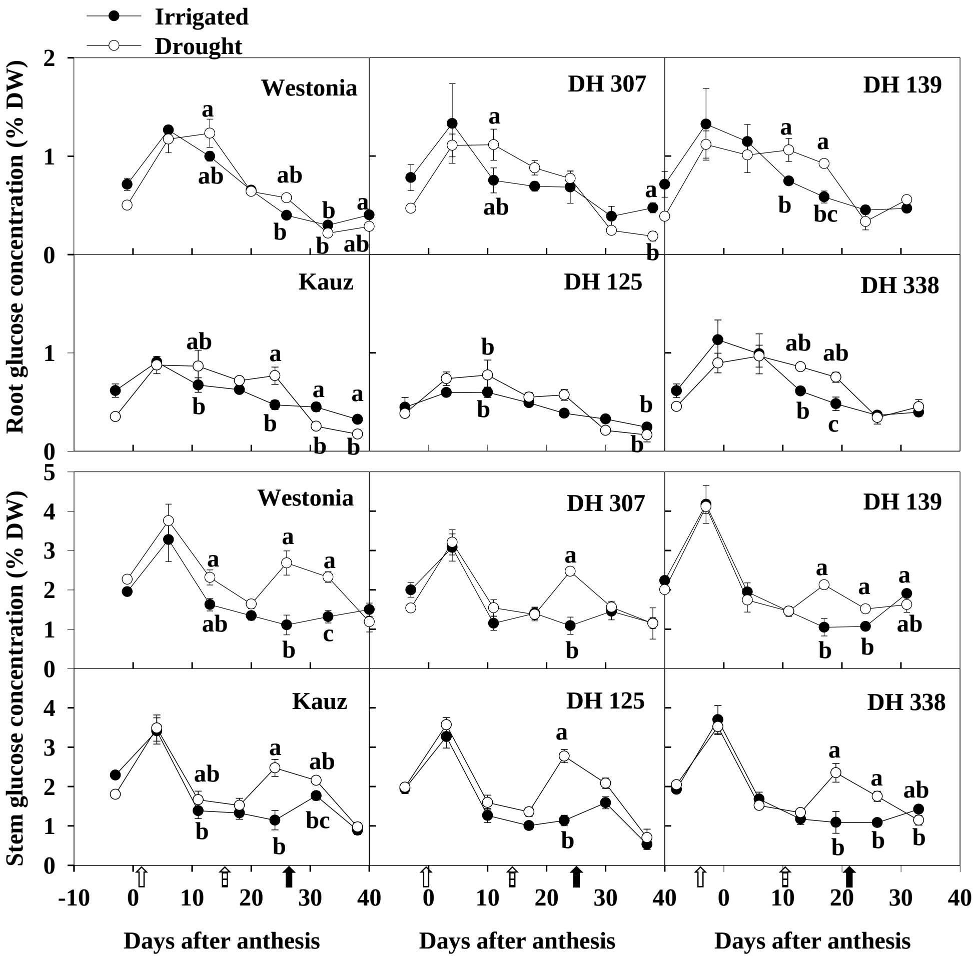

Supplementary Figure S6. The levels of glucose in roots and stems of DH 307, DH 125, DH 139, DH 338 and the parental lines of Westonia and Kauz under drought and irrigated conditions in the field, respectively. The vertical bars represent SE. Values with the same letter are statistically not different at $P = 0.05$. Open, dashed and closed arrows indicate start of drought treatment, 6 mm and 10 mm of rainfall, respectively
